# Geographic variation in access among adults with kidney disease: evidence from medical expenditure panel survey, 2002–2011

**DOI:** 10.1186/s12913-016-1844-1

**Published:** 2016-10-18

**Authors:** Mukoso N. Ozieh, Kinfe G. Bishu, Rebekah J. Walker, Jennifer A. Campbell, Leonard E. Egede

**Affiliations:** 1 Center for Health Disparities Research, Department of Medicine, Medical University of South Carolina, Charleston, SC USA; 2 Department of Medicine, Division of General Internal Medicine and Geriatrics, Medical University of South Carolina, Charleston, SC USA; 3 Health Equity and Rural Outreach Innovation Center (HEROIC), Ralph H. Johnson Veterans Affairs Medical Center, Charleston, SC USA

**Keywords:** Geographic variation, Kidney disease, Access to care, MEPS

## Abstract

**Background:**

To understand geographic variation in access to care over time in patients with kidney disease.

**Methods:**

We analyzed 4404 (weighted sample of 4,251,129) adults with kidney disease from the United States using the Medical Expenditure Panel Survey over 10 years. Three dependent variables were created to investigate variation in access: usual source of care, overall medical access to care, which took into account usual source of care, ability to get care, and delay in care, and prescription access, which took into account ability to get prescriptions and delay in getting prescriptions. Multiple logistic regression was used with geographic region as the main independent variable, adjusting for relevant covariates.

**Results:**

Compared to the Northeast region, adults living in the Midwest (OR = 0.56; 95 % CI 0.35–0.89), South (OR = 0.48; 95 % CI 0.32–0.72) and West (OR = 0.53; 95 % CI 0.34–0.84) had significantly lower odds of reporting usual source of care. For the combined access measure, compared to Northeast, adults in Midwest (OR = 0.60; 95 % CI 0.40–0.88), South (OR = 0.62; 95 % CI 0.44–0.88) and West (OR = 0.50; 95 % CI 0.34–0.72) had significantly lower odds of medical access to care. Region was not significantly associated with the odds of having prescription access, though a significant increase in prescription access was observed over time.

**Conclusions:**

Geographic variation in access to care among adults with kidney disease exists independent of income, education, insurance and comorbid conditions, with those in the South least likely to have a usual source of care and those in the West least likely to have overall access to care when compared to the Northeast United States.

## Background

A fundamental link between healthcare systems and the populations they serve is access to care, which is a multidimensional concept concerned with determining whether those who need care can enter the healthcare system [1–3]. The Institute of Medicine defined access as “a timely use of personal health services to achieve the best possible health outcomes” [4]. While actual access can be gauged by health care utilization information, measures gathering information on the ability to receive care when it is needed may be more appropriate [2, 3]. As a result, global measures of access, such as having a primary care provider, ability to obtain care when needed, or delay in needed medical care, help evaluate overall access to care over time [3].

Studies have identified barriers to care to include financial, psychological, social, organizational, spatial, and temporal factors; and differences by social characteristics and enabling resources may indicate inequitable care [3, 5]. Investigations note inequity in access for low-income and minority populations including irregular source of care, lack of preventative care, and delay in obtaining needed care [3, 4]. However, fewer studies investigate regional differences in care. As region is an immutable personal factor in healthcare, variations in this measure offer insight on system-level factors that may need targeted interventions [2]. An analysis using data from the Medical Expenditure Panel Survey (MEPS) found regional differences in care for the general population. Americans in the West and South were less likely to have a usual source of health care than those in the Northeast and Midwest [1]. It also revealed that those in the Northeast were the least likely to encounter difficulty or delay in obtaining medical care [1]. Large regional variations were also seen in the proportion of Americans that report having a regular doctor and those diagnosed with a chronic disease that report visiting their doctors in the past 2 years [6]. As regional accessibility involves social dimensions of access beyond constraints due to distance or travel, it is important to investigate and better understand differences in access to care [7].

Chronic medical diseases, such as kidney disease, are particularly important to consider in an analysis of access to care because those with a usual source of care are more likely to obtain treatment for chronic health conditions [1]. However, though those disabled with end-stage renal disease (ESRD) receive coverage through Medicare, disparities in outcomes and access to quality care continue [8]. For instance, socioeconomic and or geographic variation has been reported in Medicare spending for people with ESRD and in the kidney transplant population, and racial/ethnic minorities have a greater burden and worse outcomes for chronic kidney disease (CKD) [9–13]. Eliminating barriers to care for those with kidney disease or risk factors for kidney disease continues to be a concern. Specifically, there is a need to provide access to care in order to monitor pre-dialysis care and prevent progression of disease [8, 14, 15]. Studies have found differences in access by poverty status, incidence and awareness of disease, and variation in Medicaid coverage; however, most studies focus on understanding disparities in kidney transplantation rather than kidney disease [8, 10, 16–18]. As a result, the association between geographic variation and access to care over time in patents with kidney disease remains unclear. To our knowledge, no study has used national data over a prolonged time period to examine geographic variation in access among adults with kidney disease.

Therefore, the aim of this study is to understand geographic variation in access to care over time in patients with kidney disease. In an effort to understand changes over time and inform policy decisions, this analysis uses global definitions for access, broadens the disease classification beyond only CKD or ESRD, and uses national data to allow for generalizable estimates.

## Methods

### Data source and sample

We analyzed the responses of 4404 adults (≥18 years) with kidney disease from the United States using the Medical Expenditure Panel Survey (MEPS) for the years 2002–2011. MEPS is a nationally representative survey of the U.S. civilian non-institutionalized population and is administered by the Agency for Healthcare Research and Quality [19, 20]. MEPS obtains comprehensive information on participants’ use of medical care, prescription medication and their medical spending, as well as information on demographics, socioeconomics and satisfaction with health care. The complex survey design includes multistage sampling, clustering and stratification with oversampling of minorities [21]. Using weights provided by the Agency for Healthcare Research and Quality to account for sampling, the weighted sample for this analysis represented 4,251,129 adults with kidney disease living in the United States.

Self-reported information is collected from respondents, in addition to collection of data on medical and financial variables from all types of health care providers for validation and supplementation [19]. Diagnosis coded according to International Classification of Disease, Ninth Revision, Clinical Modification (ICD-9-CM) are also collected. Kidney disease related medical conditions and procedures reported by respondents were recorded by an interviewer as verbatim and then converted by professional coders to ICD-9-CM codes. Fully specified ICD-9-CM codes were collapsed into three digits in order to protect confidentiality of respondents [20]. The error rate for any coder did not exceed 2.5 % on verification [22]. For each year, data were merged from the medical condition files and the full-year consolidated files using the unique person identifier (DUPERSID) on a one-to-one match [20]. To ensure sufficient sample size and robust estimation for our analysis [23–25], we pooled the 10-year MEPS data.

### Measures

All measures are based on previously validated questionnaires that are publicly available on the MEPS website [19, 20]. Individuals with kidney disease were identified from the MEPS household medical condition files using clinical classification categories (CCCs) codes of 156 (nephritis, nephrosis, renal sclerosis), 157 (acute and unspecified renal failure), 158 (chronic renal failure),160 (calculus of urinary tract) and 161 (other diseases of kidney and ureters) [20].

The dependent variables in this study were dichotomized variables created from questions asked in the access section of MEPS. The first access variable was Usual Source of Care, as determined by response to the question: Do you have a usual source of care provider? An overall Medical Access to Care variable was created from responses to three separate questions: 1) Do you have a usual source of care provider?, 2) Were you unable to get necessary medical care?, and 3) Were you delayed in getting necessary medical care?. If respondents answered ‘yes’ to having a usual source of care, ‘no’ to being unable to get necessary care, and ‘no’ to having a delay in necessary medical care they were coded as having Medical Access. Opposite answers to any of the three questions resulted in being coded as not having Medical Access. An overall Prescription Access to Care variable was created from responses to two questions: 1) Were you unable to get necessary prescription medication?, and 2) Were you delayed in getting necessary prescription medication? [20]. Similarly to Medical Access, if respondents answered ‘no’ to being unable to get prescriptions, and ‘no’ to having a delay in necessary prescriptions they were coded as having Prescription Access. Opposite answers to either of the two questions resulted in being coded as not having Prescription Access.

The primary independent variable was census region coded as: Northeast, Midwest, South and West.

### Covariates

All covariates used for analysis were based on self-report and were included to take into account sociodemographic differences between the regions. Binary indicators of co-morbidities were based on a positive response to a question “Have you ever been diagnosed with…?”. Cardiovascular disease (CVD), however, was a positive response to diagnosis with coronary heart disease, angina, myocardial infarction, or other heart diseases. Race/ethnic groups are categorized as: Non-Hispanic White (NHW), Non-Hispanic Black (NHB), Hispanic or others. Education was categorized as: less than high school (≤ grade 11), high school (grade 12) and college or more (grade ≥ 13). Marital status was coded as: married, non-married (widowed/divorced/separated) and never married. Gender was dichotomized and age was coded into three age groups: 18–44, 45–64 and ≥ 65 years. Metropolitan Statistical Areas (MSA) was dichotomized based on population as of end of the year. Health insurance was coded as: private, public only and uninsured at all time in the year. The income level was defined as a percentage of the poverty level and grouped in to four categories: poor (<125 % of poverty level), low income (125 % to less than 200 % of poverty level), middle income (200 % to less than 400 % of poverty level) and high income (≥400 % of poverty level). Calendar year was grouped into 2002/03, 2004/05, 2006/07, 2008/09, 2010/11 for the pooled data.

### Statistical analysis

We used multiple logistic regressions with binary variables of Usual Source of Care, Medical Access to Care, and Prescription Access to Care as the dependent variables (yes versus no) across geographic region, adjusting for age, sex, race/ethnicity, marital status, education, insurance status, MSA status, household income, comorbidities and calendar year. For interpretation, we use the adjusted odds ratio coefficient of the logistic regression.

F-adjusted mean residual goodness-of-fit was applied to test the adequacy of the models. After fitting the logistic regression models taking the survey design in to account, the F-adjusted mean residual goodness-of-fit suggested no evidence of lack of fit [26]. The link test that account complex survey design, used as a diagnostic test to examine the model specification error, verified no evidence of model specification error in the models [27]. Using the Variance inflation factor (VIF) test, and taking into account the complex survey design, it was determined that no multicollinearity problems existed between predictors of the models. All analyses were performed at the person-level using STATA 14 (StataCorp LP College Station, TX). Only estimates that are statistically significant at the *p* < 0.05 level are discussed.

## Results

### Characteristics of U.S. Adults with kidney diseases (KD)

Characteristics of adults with kidney diseases (KD) in the study are summarized in Table 1. Of the sample population representing 4404 U.S. adults with KD, 15.5 % were from Northeast, 19.7 % were from Midwest, 42.5 % were from South and the remaining 22.3 % were from the West region. Non- Hispanic Whites with KD were more likely in the Midwest region (83.2 compared to 73.6 % mean), Non-Hispanic Blacks with KD were more likely in the South region (14.4 compared to 11.3 % mean), and Hispanic and Others with KD were more likely in the West region (22.9 compared to 11.1 % mean for Hispanics, and 9.1 compared to 4.0 % mean for Other) (*p* < 0.001). Privately insured adults with KD were least likely in the West (57.4 compared to 64.4 % mean), whereas publicly insured adults with KD were more likely in the West (33.3 compared to 28.2 % mean), and uninsured adults with KD were more likely in the South and West regions (9.3 for both compared to 7.4 % mean) (*p* < 0.001). Poor and low income individuals with KD were more likely in the Midwest region (22.4 compared to 19.6 % mean), while high income individuals with KD were more likely in the Northeast region (41.3 compared to 36.3 % mean) (*p* < 0.001).

**Table 1:** Sample demographics by geographical region among U.S. adults with kidney disease (KD)

Variables	All (%)	Northeast (%)	Midwest (%)	South (%)	West (%)	*P*-value
*N* (*n*)	4,251,128 (4404)	797,487 (683)	880,779 (869)	1,746,049 (1870)	826,813 (982)	
Age category						
Age 18–44	29.6	29.7’	30.1	29.7	29.0	0.799
Age 45–64	36.6	38.7	37.5	36.0	34.5	
Age 65–85	33.8	31.6	32.4	34.3	36.5	
Gender						
Male	51.2	49.8	52.8	51.2	50.9	0.885
Female	48.8	50.2	47.2	48.8	49.1	
Race/ethnicity						
Non-Hispanic White	73.6	75.9	83.2	73.0	62.5	<0.001
Non-Hispanic Black	11.3	9.7	11.8	14.4	5.5	
Hispanic	11.1	10.6	3.1	9.8	22.9	
Other	4.0	3.8	1.9	2.8	9.1	
Marital status						
Married	56.9	57.4	60.5	55.1	56.5	0.254
Non-married ^a^	28.6	25.0	27.0	30.7	29.5	
Never married	14.5	17.6	12.5	14.2	14.0	
Education category						
< High school	21.9	17.8	20.4	24.2	22.7	0.011
High school	33.1	40.3	35.1	30.3	29.8	
College or more	45.0	41.9	44.5	45.5	47.5	
Insurance						
Private	64.4	68.9	67.5	64.0	57.4	<0.001
Public	28.2	26.1	28.3	26.7	33.3	
Uninsured	7.4	5.0	4.2	9.3	9.3	
Metropolitan statistical status						
MSA	81.3	86.3	73.1	81.1	85.6	0.014
Non-MSA	18.7	13.7	26.9	18.9	14.4	
Poverty category						
Poor/NEA	19.6	18.5	16.9	20.3	22.4	<0.001
Low Income	15.5	14.1	17.7	15.1	15.2	
Middle Income	28.6	26.1	29.6	29.0	28.8	
High Income	36.3	41.3	35.8	35.6	33.6	
Chronic conditions						
Diabetes	28.9	25.6	28.3	29.6	31.5	
Hypertension	56.1	52.2	56.2	56.6	59.0	0.347
CVD	30.4	28.3	29.9	30.6	32.3	0.679
Stroke	8.4	7.9	8.3	7.8	10.1	0.642
Emphysema	4.1	3.2	3.2	4.7	4.3	0.457
Joint pain	51.0	44.6	51.9	52.8	52.6	0.022
Arthritis	41.9	39.9	44.1	43.5	38.2	0.162
Asthma	11.5	11.0	10.0	11.9	12.8	0.521
Year category						
Year 2002/03	17.7	18.4	18.9	17.6	16.0	0.901
Year 2004/05	18.4	17.3	19.6	18.5	17.8	
Year 2006/07	19.3	20.9	19.1	18.6	19.5	
Year 2008/09	20.9	22.2	20.6	20.0	22.0	
Year 2010/11	23.7	21.2	21.8	25.3	24.7	
Access						
Usual source of care	89.7	93.4	90.6	88.1	88.8	0.004
Medical access	82.1	88.1	82.6	81.2	77.9	<0.001
Prescription access	91.8	94.4	92.2	90.8	90.8	0.064

N-weighted sample size; n-unweighted sample size; %, weighted percentage

^a^Non-married stands for widowed/divorced and separated

### Access to care

Table 2 presents the results from the logistic regression of whether adults with KD indicated usual source of care. In Table 3, we report our findings from logistic regression of whether adults with KD had medical access to care, as defined in the methods. Table 4 shows the logistic regression of whether adults with KD had prescription access.

**Table 2:** Logistic regression model for usual source of care among adults with kidney disease (KD) by geographical status

Variables	Odds ratio	95 % CI	*p* value
Primary independent variables			
Northeast (ref)	–	–	–
Midwest	0.56*	0.35–0.89	0.016
South	0.48**	0.32–0.72	0.001
West	0.53**	0.34–0.84	0.007
Covariates			
Age			
Age 18–44 (ref)	–	–	–
Age 45–64	2.0***	1.48–2.85	<0.001
Age 65–85	2.95***	1.96–4.43	<0.001
Gender			
Female	1.43**	1.10–1.87	0.008
Race/ethnicity			
Non-Hispanic White (ref)	–	–	–
Non-Hispanic Black	0.92	0.58–1.48	0.753
Hispanic	0.71	0.49–1.02	0.071
Others	0.65	0.38–1.11	0.119
Marital Status			
Married (ref)	–	–	–
Not married ^a^	0.64*	0.45–0.93	0.019
Never married	0.61**	0.44.–0.84	0.003
Education			
< High school (ref)	–	–	–
High school	0.86	0.60–1.22	0.403
College or more	0.82	0.58–1.15	0.262
Insurance Status			
Private (ref)	–	–	–
Public insured	1.02	0.70–1.49	0.909
Uninsured	0.28***	0.20–0.40	<0.001
MSA status			
MSA	1.10	0.79–1.55	0.544
Poverty Category			
Poor/NEA (ref)	–	–	–
Low Income	1.13	0.74–1.72	0.543
Middle Income	1.33	0.93–1.92	0.115
High Income	1.44	0.94–2.18	0.086
Chronic Conditions ^b^			
Diabetes	1.47*	1.02–2.12	0.034
Hypertension	2.08***	1.53–2.81	<0.001
CVD	1.56**	1.09–2.21	0.014
Stroke	1.05	0.49–2.23	0.896
Emphysema	0.37*	0.16–0.86	0.022
Joint Pain	1.12	0.83–1.51	0.444
Arthritis	1.38	0.97–1.97	0.071
Asthma	1.35	0.89–2.05	0.148
Year			
Year 2002/03 (ref)	–	–	–
Year 2004/05	0.93	0.62–1.41	0.766
Year 2006/07	0.72	0.48–1.08	0.117
Year 2008/09	0.65*	0.43–0.97	0.039
Year 2010/11	0.80	0.52–1.22	0.306

*Level of significance *p* < 0.05; **level of significance *p* < 0.01, ***level of significance *p* < 0.001

^a^Non-married stands for widowed/divorced and separated

^b^Reference for each chronic conditions was not having that specific condition

**Table 3:** Logistic regression model for medical access to care among adults with kidney disease (KD) by geographical status

Variables	Odds ratio	95 % CI	*p* value
Primary independent variables			
Northeast (ref)	–	–	–
Midwest	0.60**	0.40–0.88	0.009
South	0.62**	0.44–0.88	0.008
West	0.50***	0.34–0.72	<0.001
Covariates			
Age			
Age 18–44 (ref)	–	–	–
Age 45–64	1.80***	1.39–2.32	<0.001
Age 65–85	2.70***	1.91–3.82	<0.001
Gender			
Female	1.22*	1.0–1.48	0.048
Race/ethnicity			
Non-Hispanic White (ref)	–	–	–
Non-Hispanic Black	1.10	0.78–1.54	0.578
Hispanic	0.99	0.72–1.36	0.975
Others	0.55**	0.35–0.85	0.009
Marital Status			
Married (ref)	–	–	–
Not married ^a^	0.65**	0.50–0.85	0.002
Never married	0.62**	0.47–0.83	0.001
Education			
< High school (ref)	–	–	–
High school	0.95	0.72–1.26	0.769
College or more	0.91	0.68–1.21	0.542
Insurance Status			
Private (ref)	–	–	–
Public insured	1.04	0.79–1.37	0.754
Uninsured	0.22***	0.16–0.30	<0.001
MSA status			
MSA	1.0	0.76–1.33	0.945
Poverty Category			
Poor/NEA (ref)	–	–	–
Low Income	1.11	0.78–1.56	0.547
Middle Income	1.25	0.93–1.68	0.124
High Income	1.46*	1.05–2.03	0.024
Chronic Conditions ^b^			
Diabetes	1.0	0.74–1.34	0.749
Hypertension	1.52***	1.20–1.93	<0.001
CVD	1.08	0.85–1.38	0.510
Stroke	0.88	0.59–0.94	0.437
Emphysema	0.70	0.39–1.26	0.241
Joint Pain	0.75*	0.59–0.94	0.014
Arthritis	1.19	0.92–1.54	0.166
Asthma	0.92	0.67–1.26	0.626
Year			
Year 2002/03 (ref)	–	–	–
Year 2004/05	0.99	0.70–1.40	0.975
Year 2006/07	0.93	0.66–1.32	0.715
Year 2008/09	0.95	0.68–1.31	0.770
Year 2010/11	0.88	0.63–1.24	0.491

*Level of significance *p* < 0.05; **level of significance *p* < 0.01, ***level of significance *p* < 0.001

^a^Non-married stands for widowed/divorced and separated

^b^Reference for each chronic conditions was not having that specific condition

**Table 4:** Logistic regression for prescription access among adults with kidney disease (KD) by geographical status

Variables	Odds ratio	95 % CI	*p* value
Primary independent variables			
Northeast (ref)	–	–	–
Midwest	0.77	0.47–1.24	0.284
South	0.68	0.46–1.0	0.051
West	0.64	0.40–1.02	0.064
Covariates			
Age			
Age 18–44 (ref)	–	–	–
Age 45–64	1.21	0.83–1.76	0.310
Age 65–85	2.6***	1.65–4.06	<0.001
Gender			
Female	0.85	0.64–1.13	0.278
Race/ethnicity			
Non-Hispanic White (ref)	–	–	–
Non-Hispanic Black	1.13	0.80–1.61	0.463
Hispanic	1.5*	1.0–2.18	0.049
Others	0.97	0.51–1.82	0.934
Marital Status			
Married (ref)	–	–	–
Not married ^a^	0.57**	0.39–0.82	0.003
Never married	0.84	0.54–1.29	0.436
Education			
< High school (ref)	–	–	–
High school	1.26	0.87–1.81	0.209
College or more	1.43	0.98–2.09	0.059
Insurance Status			
Private (ref)	–	–	–
Public insured	0.77	0.53–1.11	0.164
Uninsured	0.36***	0.23–0.56	<0.001
MSA status			
MSA	0.95	0.66–1.36	0.807
Poverty Category			
Poor/NEA (ref)	–	–	–
Low Income	1.14	0.75–1.72	0.519
Middle Income	1.14	0.77–1.70	0.500
High Income	1.22	0.75–1.96	0.408
Chronic Conditions ^b^			
Diabetes	0.67*	0.50–0.91	0.011
Hypertension	0.72	0.51–1.0	0.051
CVD	0.80	0.57–1.13	0.214
Stroke	1.06	0.70–1.61	0.763
Emphysema	0.88	0.47–1.65	0.705
Joint Pain	0.55***	0.39–0.76	<0.001
Arthritis	1.05	0.75–1.45	0.759
Asthma	0.51**	0.35–0.75	0.001
Year			
Year 2002/03 (ref)	–	–	–
Year 2004/05	1.30	0.87–1.94	0.193
Year 2006/07	1.65*	1.08–2.52	0.019
Year 2008/09	2.02**	1.32–3.10	0.001
Year 2010/11	1.50	0.96–2.35	0.074

*Level of significance *p* < 0.05; **level of significance *p* < 0.01, ***level of significance *p* < 0.001

^a^Non-married stands for widowed/divorced and separated

^b^Reference for each chronic conditions was not having that specific condition

Compared to the Northeast region, adults living in the Midwest (OR = 0.56; 95 % CI 0.35–0.89), South (OR = 0.48; 95 % CI 0.32–0.72) and West (OR = 0.53; 95 % CI 0.34–0.84) had significantly lower odds of reporting a usual source of care. Other covariates significantly associated with usual source of care included higher age, (aged 45–64 (OR = 2.0; 95 % CI 1.48–2.85) and aged 65–85 (OR = 2.95; 95 % CI 1.96–4.43), female gender (OR = 1.43; 95 % CI 1.10─1.87). Covariates significantly associated with less usual source of care included uninsured (OR = 0.28; 95 % CI 0.20–0.40). Comorbid conditions like diabetes (OR = 1.47; 95 % CI 1.02–2.12), hypertension (OR─2.08; 95 % CI 1.53–2.81) and CVD (OR = 1.56; 95 % CI 1.09–2.21) had a higher odds but emphysema had a lower odds (OR = 0.37; 95 % CI 0.16–0.86) of having usual source of care. Compared to the bench mark year of 2002/03, calendar year 2008/09 had a lower odds of having a usual source of care (OR = 0.65; 95 % CI 0.43–0.97).

When considering the combined access measure, compared to Northeast region, adults in Midwest (OR = 0.60; 95 % CI 0.40–0.88), South (OR = 0.62; 95 % CI 0.44–0.88) and West (OR = 0.50; 95 % CI 0.34–0.72) had significantly lower odds of medical access to care. Again more access was associated with age (aged 45–64 (OR = 1.80; 95 % CI.39–2.32) and aged 65–85 (OR–2.70; 95 % CI 1.91 – 3.82)), and female gender (OR = 1.22; 95 % CI 1.0–1.48). Other race/ethnicity had a lower odds of medical access (OR = 0.55; 95 % CI 0.35–0.85) compared with their non-Hispanic white counterparts, as did uninsured adults (OR = 0.22; 95 % CI 0.16–0.30) compared with privately insured groups. Higher income groups had statistically significant higher odds of medical access (OR = 1.46; 95 % CI 1.05–2.03). Adults with comorbid hypertension had higher odds of medical access (OR = 1.52; 95 % CI 1.20–1.93), but joint pain had a lower odds (OR = 0.75; 95 % CI 0.59–0.94) compared to their counter parts.

Region was not significantly associated with the odds of having prescription access, and compared to 2002/03, both calendar years (2006/07 and 2008/09) were associated with higher odds of prescription access.

## Discussion

The results of this study demonstrate geographic variation in access to care in patients with kidney disease over time, with a significant decrease in having a usual source of care in 2008/09 compared to 2002/03. Access to prescriptions showed no geographic variation, however, there was a significant increase in access to prescription medications across the years investigated. Amongst adults with kidney disease, living in the South, West, and Midwest is associated with a lower likelihood of usual source of care and lower overall access to care compared to living in the Northeast. Additionally, adults with kidney disease with comorbid diabetes, hypertension, and CVD had 1.5 to 2 times higher likelihood of usual source of care whereas those with comorbid emphysema had a lower likelihood of usual source of care.

To our knowledge, this study is the first of its kind to investigate trends in geographical variation in access to care over a decade using a nationally representative sample among adults with kidney disease. While prior research has established geographic variation in access to care among patients with ESRD and kidney transplantation [10, 16, 17], this study demonstrates disparate access to care for patients with any diagnosis of kidney disease. This suggests that poor outcomes for those with CKD and ESRD may be linked to barriers to access occurring early in the disease process. Lack of regular access to care may serve to compound disease burden and result in the early onset of disease related complications. For instance, as reported by the US Renal Data System, nearly half of the patients who started receiving treatment for CKD never received care from a nephrologist prior to CKD onset [28]. While barriers to care are multidimensional and can be influenced at the patient level by disease awareness and patient preference; the geographic variation in access, as well as outcomes, suggest that system level barriers to access may have an impact on disease complications and outcomes through a patient’s ability to obtain care.

In this analysis we investigated three related, but conceptually separate measures of access to care: having a usual source of care, a more comprehensive measure of access incorporating considerations including delay of care, and having access to prescriptions. As access is a complex construct, it is important to consider all three to have a better understanding of overall access to care for patients with kidney disease. These results suggest that while there are no geographic differences in access to prescriptions, differences do exist in access to medical care, whether using a more basic measure such as usual source of care, or a more comprehensive measure. In addition, while in both medical access measures, those with kidney disease in the Northeast were more likely to have access, the relative differences between other regions and the Northeast differed between the two measures. While those in the South had the lowest odds of having a usual source of care, it was those in the West that had the lowest odd of having access when taking ability to obtain care, and not having a delay in care into account. These results support findings of a commonwealth fund report that revealed geography influences access to care and quality of care [29]. Similar to our study, they found that areas in the Northeast and Midwest had higher measures of access compared to areas in the South. They also found strong relationships between access to care and outcomes, with better access being associated with better outcomes. In the Southeast region, it has also been reported that testing and detection effort are low compared to other regions [30]. Unfortunately, this at-risk region (the Southeast) has the highest prevalence of recognized CKD, further suggesting the need to examine the impact that system level barriers may have in the variation in access for patients with kidney disease and those at risk for CKD and ESRD.

The Healthy People 2020 objective for chronic kidney disease (CKD) is to reduce new cases of CKD and its complications, disability, death and economic costs [31]. Access to care ensures at-risk individuals get timely, preventative and recommended care so as to obviate when possible the complications and economic burden of kidney disease. Lack of access to care not only affects an individual’s ability to maintain their health, but also negatively impacts quality of life as a whole. Therefore, understanding geographic variation in access is vital for establishing successful policies geared towards reducing the incidence, prevalence and complications of kidney disease across the nation [30]. The Affordable Care Act (ACA) is one of the most recent national undertakings to improve access to care. The ACA—Medicaid expansion was implemented as part of this act to improve access for low income Americans, but this mandate is dependent on state implementation [32]. Unfortunately, many of the states that have chosen not to implement this law are in at-risk regions of the USA based on this analysis, such as the Southeast [33]. This study serves as baseline information for policymakers on existing regional variation in access to care among adults with kidney disease in the midst of the Affordable Care Act (ACA) reform. Future studies should consider how the ACA-Medicaid expansion influenced variations seen.

## Conclusions

The strengths of this study include the use of nationally representative data over a prolonged period, including broad care categories (access to usual source of care, medical access care, and prescription access care) and the use of a robust estimation method to examine geographic variation in access to care accounting for comorbidities among patents with kidney disease. However, limitations do exist. First, MEPS relies on self-report, and as such may be prone to potential bias and recall error. However, it is one of the few national dataset with extensive information on health care access, utilization and cost, and questions have been validated over time to collect reliable information. Second, there is no laboratory information in MEPS and the ICD-9 codes for chronic kidney disease are collapsed for confidentiality reasons, so we are unable to examine geographic trends by CKD stage. Third, while the panel design of this study and the ability to investigate responses over different years provides trends over time, individual respondents were not followed over time, and therefore the results of our study should not be interpreted longitudinally.

This study provides insight into geographic variation in access to care among adults with kidney disease independent of income, education, insurance and comorbid conditions. Compared to the Northeast, living in the South, West, and Midwest is associated with lower likelihood of having a usual source of care or having overall access to care. Conversely, no geographic variation was seen in access to prescription medications. These findings are the first of its kind to examine trends in access in adults with kidney disease and serves as baseline for further studies evaluating the impact of potential policy reforms to access.
